# Entropy Algorithms Using Deep Learning for Signal Processing

**DOI:** 10.3390/e25010023

**Published:** 2022-12-23

**Authors:** Gwanggil Jeon

**Affiliations:** 1 Department of Embedded Systems Engineering, Incheon National University, 119 Academy-ro, Yeonsu-gu, Incheon 22012, Republic of Korea; gjeon@inu.ac.kr; 2 Energy Excellence & Smart City Lab., Incheon National University, 119 Academy-ro, Yeonsu-gu, Incheon 22012, Republic of Korea

Image and video processing operatons are significant in our life as most electronic devices, such as PCs and mobiles, are all developed by signal processing. Therefore, signal processing can be considered as a part of computer science. Information entropy is very important in signal processing, and it was developed by Claude Shannon and Harry Nyquist who defined data representations of the physical world. Nowadays, deep learning is widely used in signal processing due to its good performance. This Special Issue calls for current studies on several signal (including image and video) processing approaches that are based on information entropy and machine learning. Papers that were both applicative and theoretical in nature were welcomed (both research papers and reviews), and contributions regarding new signal processing tools for the entropy research community were also welcome. Finally, six papers were selected for this Special Issue.

In the contribution by Jia et al., “Chaotic Mapping-Based Anti-Sorting Radio Frequency Stealth Signals and Compressed Sensing-Based Echo Signal Processing Technology,” the signal-generating design principle of anti-sequential difference histogram (SDIF) arrangements was studied for the principal arrangement approach of the SDIF [1]. In addition, the authors studied a piecewise linear chaotic mechanism with interval number parameterization based on random disturbance. In addition, they presented an approach to modulate the repetition period of broadly located signal pulses by utilizing a chaotic system. This paper is concluded with experimentation proof that was conducted from the correctness of the signal design principle. From the simulation, the authors concluded three main ideas in the work.

In the contribution by Bao et al., “PCQNet: A Trainable Feedback Scheme of Precoder for the Uplink Multi-User MIMO Systems,” the authors presented a CNN-based compression network titled PCQNet to minimalize the feedback’s overhead [2]. The authors studied the consequence of the trainable compression ratios and feedback bits on the mean squared error (MMSE) between the original precoding matrices and the restored matrices. Then, they assessed the block error rates as the performance metrics of the centralized implementation with an ideal minimum MMSE transceiver. Simulation results proved that the presented method showed near-ideal results compared with other conventional approaches. 

The contribution by Wang et al., “Efficient Entropic Security with Joint Compression and Encryption Approach Based on Compressed Sensing with Multiple Chaotic Systems”, focused on a novel approach that uses compressed sensing for image compression and encryption concurrently [3]. In this work, the hash function was used to achieve the initial parameters of two chaotic maps. Then, a sparse coefficient matrix was applied from the simple image via DWTs (discrete wavelet transforms). The pixel transformation on the sparse coefficient matrix was performed by a 2D-SCLMS system. Experimental results and the analysis proved that the compressed encryption method has merits in terms of compression performance, key spaces, denoising, and sensitivity.

In the contribution by Zhao et al., “A Complex-Valued Self-Supervised Learning-Based Method for Specific Emitter Identification,” the authors present a complex self-supervised learning approach to fully examine the unlabeled examples [4]. In the authors’ previous work, they designed an ideal data augmentation tool based on communication signals to assist the contrastive conception. In this work, they included a complex-valued network during learning to enhance the strength-to-signal error. The presented method proves the generality of handling a minimum and sufficient number of sample cases across a wide range, with 10 to 400 being annotated labeled in each group. The simulation also shows promising accuracies and strength, with performance enhancements from 10% to 16% with 10-15 SNR.

In the contribution by Liu et al., “Multi-Scale Mixed Attention Network for CT and MRI Image Fusion,” the authors present a CNN-based CT and MRI image fusion approach, which uses a pictorial saliency-based plan to reserve more valuable information [5]. In this work, a multi-scale varied attention block was studied to obtain features. This block can obtain more useable information and polish the obtained features both in the spatial and channel levels. Then, a subjective saliency-based fusion plan was utilized to generate feature maps. Finally, the fused image was generated via restored blocks. The simulation results of their method produced more details, cleaner detail information, and better and more distinct contrasts when compared to conventional approaches.

In the contribution by Sun et al., “On Architecture Selection for Linear Inverse Problems with Untrained Neural Networks,” the authors attempted to broaden the application and understanding of untrained neural network priors by exploring the interaction between the choice of architecture and measurement models [6]. The authors pointed that the target applications and signal types are key factors. The authors stimulate the issue stochastically via the learning theory and provided two applicable approaches for adjusting architectural hyper-parameters. Applying experimental evaluations, they proved that the ideal hyper-parameters may change abruptly between different evaluations and can show large performance gaps when adjusted for the incorrect evaluation. Moreover, they studied the hyper-parameters that tended to be more crucial and the hyper-parameters that can produce and influence deviations from the ideal performance.
